# Analysis of the Mitochondrial Genome of a Novosvobodnaya Culture Representative using Next-Generation Sequencing and Its Relation to the Funnel Beaker Culture

**Published:** 2014

**Authors:** A. V. Nedoluzhko, E. S. Boulygina, A. S. Sokolov, S. V. Tsygankova, N. M. Gruzdeva, A. D. Rezepkin, E. B. Prokhortchouk

**Affiliations:** National Research Center “Kurchatov Institute”, Kurchatov sq. 1, 123182, Moscow, Russia; Center of Bioengineering, Russian Academy of Sciences, 60-letiya Oktyabrya Av., 7-1, 117312, Moscow, Russia; Institute for the History of Material Culture, Russian Academy of Sciences, Dvortsovaya Naberezhnaya, 18, 191186, St. Petersburg, Russia

**Keywords:** Novosvobodnaya culture, Maikop culture, haplogroup, mitochondrial DNA, sequencing, genomics

## Abstract

The Novosvobodnaya culture is known as a Bronze Age archaeological culture in the North Caucasus region of Southern Russia. It dates back to the middle of the 4th millennium B.C. and seems to have occurred during the time of the Maikop culture. There are now two hypotheses about the emergence of the Novosvobodnaya culture. One hypothesis suggests that the Novosvobodnaya culture was a phase of the Maikop culture, whereas the other one classifies it as an independent event based on the material culture items found in graves. Comparison between Novosvobodnaya pottery and Funnelbeaker (TRB) pottery from Germany has allowed researchers to suggest that the Novosvobodnaya culture developed under the influence of Indo-European culture. Nevertheless, the origin of the Novosvobodnaya culture remains a matter of debate. We applied next-generation sequencing to study ~5000-year-old human remains from the Klady kurgan grave in Novosvobodnaya stanitsa (now the Republic of Adygea, Russia). A total of 58,771,105 reads were generated using Illumina GAIIx with a coverage depth of 13.4x over the mitochondrial (mt) DNA genome. The mtDNA haplogroup affiliation was determined as V7, suggesting a role of the TRB culture in the development of the Novosvobodnaya culture and supporting the model of sharing between Novosvobodnaya and early Indo-European cultures.

## INTRODUCTION

Since the late 1970s, as archaeological evidence has accumulated, two points of view have emerged pertaining to the emergence of cultural artifacts in the Early Bronze Age in the North Caucasus. One hypothesizes the existence of a single Maikop culture with two developmental phases [1-3], including finds discovered in Novosvobodnaya stanitsa (former Tsarskaya). The other hypothesis suggests that the archaeological collections assembled in Novosvobodnaya stanitsa should be treated individually, as independent artifacts (as a distinct culture). During the archaeological excavations of the kurgan grave “Klady” near Novosvobodnaya stanitsa in 1979–1991, which were supervised by A.D. Rezepkin, a total of 22 kurgans were uncovered with 93 well-stratified burial sites. These records allow one not only to establish the absolute chronology of the artifacts, but also to contribute to a better understanding of the origin of the Novosvobodnaya culture [4, 5].

Since recently, state-of-the-art tools for genomic analysis have been widely used to solve archaeological [6-10] and paleontological riddles [11-15]. Such studies usually analyze mitochondrial DNA (mtDNA) that possesses characteristics essential for the study of human evolution: maternal inheritance; multiple copies of the mitochondrial genome; accelerated accumulation of mutations relative to the nuclear genome; no genetic recombination; a relatively high integrity of mtDNA in ancient human bone remains, since nuclear DNA breaks down twice as fast as mtDNA does [16, 17].

In this work, we report on the complete mitochondrial genome sequence of a representative of the Novosvobodnaya culture. Our findings suggest that this culture should be related to the Funnel Beaker culture. DNA testing has shown that the human sample from the Novosvobodnaya archaeological site belongs to mtDNA haplogroup V7.

## EXPERIMENTAL

**Fig. 1:**
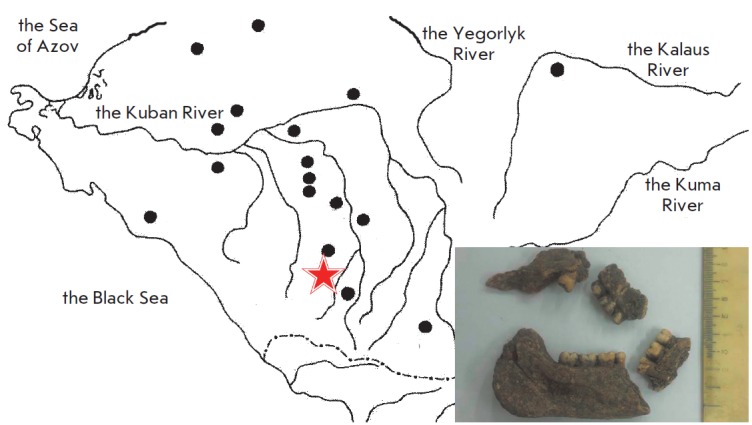
The range covered by the Novosvobodnaya culture in the North Caucasus (marked with black dots). The site of archaeological excavations of the Klady kurgan near Novosvobodnaya stanitsa in Adygeya (marked with an asterisk) and the bone remains used for the DNA analysis

DNA was extracted from tooth remains discovered during excavations of the kurgan grave “Klady” near Novosvobodnaya stanitsa (The Republic of Adygea) as part of the expedition organized by the Institute for the History of Material Culture, Russian Academy of Sciences (St. Petersburg). The remains date back to approximately Middle to Late 4000 BC (*Fig. 1*).

**DNA extraction, preparation of DNA libraries, and sequencing**

Ancient DNA was recovered from bone powder under carefully controlled laboratory conditions to avoid contamination with modern human DNA. Proteinase K (New England Biolabs, USA) and silica beads (Sigma- Aldrich, USA) were used as previously described by Orlando *et al*. [18]. DNA libraries were prepared using a NE BNext Quick DNA Library Prep Master Mix set for 454 (New England Biolabs) with adapter primers on an Illumina Sequencing Platform following the manufacturer’s instructions. The purity and amount of DNA libraries were evaluated using a 2100 Bioanalyser (Agilent, USA) and HS Quibit (Invitrogen, USA). For the enrichment of mtDNA, a FleXelect Mitochondrial DNA enrichment kit was utilized (Flexgen, Netherlands) with probes overlapping by 10 to 40% (a detailed list of the oligonucleotide probes for mtDNA enrichment is available upon request). The DNA libraries were sequenced using 50 bp paired-end reads on an Illumina GAIIx instrument.

**Bioinformatics analysis**

The reads were mapped against the mitochondrial reference sequence (NC _012920.1) using Bowtie2 version 2.1.0 with the *very-sensitive *option [19]. Ancient DNA sequences were authenticated with mapDamage 2.0 [20]. According to the model of post-mortem damage inferred using this software, the quality score was adjusted to allow for nucleotide mismatches. Alternatively, nucleotides that may have arisen from C → T or G → A substitutions were given the lowest quality scores than in the original reads and removed from the analysis of the single nucleotide polymorphisms (SNPs) exhibited by the sample. SNPs were searched for using the VarScan software (v 2.2.3) and selected at *p * < 0.01 [21]. The mtDNA haplogroup affiliation was determined based on the SNPs with the HaploGrep webtool [22]. *De novo *assembly of the mitochondrial genome of a representative of the Novosvobodnaya culture was conducted using AbySS version 1.3.6. at a k-mer of 22 nucleotides [23].

## RESULTS AND DISCUSSION

**Fig. 2:**
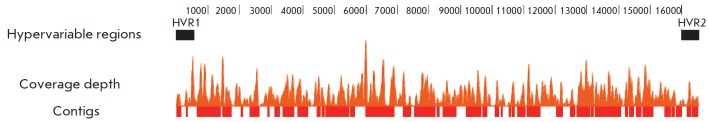
The results of mitochondrial genome sequencing and de novo assembly

Mitochondrial DNA sequences were retrieved from the libraries of ancient human DNA. A total of 58,771,105

reads were produced from enriched libraries, most part of which (99.994%) consisted of environmental DNA sequences (bacterial), which commonly occurs in an ancient DNA analysis [24], or could be explained by the relatedness between bacterial and eukaryotic mitochondrial genes. Read mapping against the reference mitochondrial genome sequence (hg19) allowed us to achieve a coverage depth of 13.4X: a total of 3,422 reads were uniquely mapped (0.006%) (*Fig 2*).

**Fig. 3:**
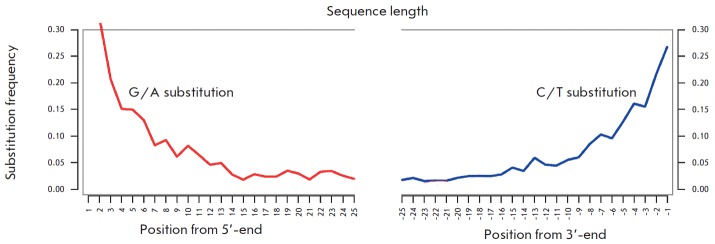
Nucleotide misincorporation patterns and contamination test

Ancient DNA is known to degrade into short fragments over time; cytosine residues (C) located at the ends deaminate to uracil (U) and turn into thymine (T) during sample preparation (PCR ). The frequency of terminal C → T substitutions in samples dated older than 300 thousand years could be up to 60% and higher [15, 26]. At the same time, sequencing of modern DNA demonstrates less than 0.5% terminal nucleotide substitutions (data not shown). The substitution frequency was calculated using MapDamage 2.0. The frequency of C → T substitutions at the 3'- and 5'-ends of the DNA libraries exceeded 30% in the sample from Novosvobodnaya stanitsa (*Fig. 3*). This finding argues for the fact that the total mtDNA is of ancient origin.

**Fig. 4:**
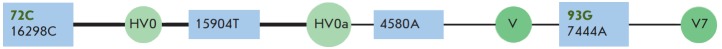
Mitochondrial haplogroup assignment. The tree branch generated by HaploGrep service is shown. SNPs in green and blue rectangles (72C and 93G) were used for haplogroup determination

When compared with the consensus mitochondrial genome sequence (in light of lowering quality scores for terminal substitutions, see the Experimental section), individual reads from the sample of an ancient human of Novosvobodnaya stanitsa yielded SNPs, indicating an affiliation within Haplogroup V7 (*Fig. 4*, *Table*).

**Table:** SNPs (p < 0.01) discovered in the Novosvobodnaya mitochondrial genome

mtDNA SNP position	mt DNA reference sequence (hg19)	A substitution in the sample	Gene
72*	T	C	-
93*	A	G	-
2515	C	T	TVAS5
9378	G	A	JA760602; JA760600
9541	C	T	JA760602; JA760600
11018	C	T	JA760602; JA760600; STR F6; JA760615
11720	A	G	JA760602; JA760600; STR F6; JA760615
11723	C	T	JA760602; JA760600; STR F6; JA760615
12851	G	A	JA760602; JA760600; JA760615
14906	A	G	JA760602; cytochrome b
15302	A	G	JA760602; cytochrome b
15477	C	T	JA760602; cytochrome b

* – SNPs used for haplogroup V7 assignment

The assembly of the mitochondrial genome of a human from the Novosvobodnaya culture performed with a minimum contig length of 100 nucleotides produced a N50 contig length of 203 nucleotides (N50 is the maximum contig length in *de novo *assembly, such that 50% of the entire assembly is contained in contigs equal to or longer than the N50 length).

The total *de novo *assembly of mtDNA generated 11,063 nucleotides (*Fig. 2*). Because of ancient DNA degradation*, *asymmetric PCR amplification and enriched mtDNA (FleXelect Mitochondrial DNA enrichment kit), most contigs were short and did not overlap, which prevented the assembly of the contigs into a complete mtDNA sequence despite the coverage depth of 13.4X.

Recently, archaeological evidence has emerged to argue against the opinion that the Novosvobodnaya culture shares links with the West Asian Maikop culture. The discovered artifacts support the hypothesis that the Baalberg phase of early periods of the Indo*-*European Funnel*-*Beaker culture played a significant role in the Novosvobodnaya archaeological culture, rather than the West Asian Maikop culture [5]. To prove or rule out this hypothesis, a DNA analysis is required as one of the definitive tools.

Genetic studies devoted to ancient human migrations across Europe have been extensive in the past decades as reviewed by B. Sykes [27]. Thus, in Europe the major mtDNA haplogroups were U, H, V, I, W, T, and K, which appeared and spread 11–14 thousand years ago during de-glaciation. Haplogroup J may have arrived from the Middle East during an influx of farmers [27]. Sequencing of the mtDNA of representatives of the Linear Pottery culture (the ancestor of the Funnel Beaker culture) allowed one to identify the dominant haplogroups as H, V, and T [28]. In addition, some studies have demonstrated that during the time of the Linear Pottery and related cultures, the haplogroups U, H, and V predominantly occurred in Europe [29-32]. Our findings, obtained using current genetic analysis techniques, are in agreement with the hypothesis of the origin of the Novosvobodnaya culture proposed by A.D. Rezepkin [5].

## CONCLUSIONS

We have reported on the sequencing of the mtDNA genome of an ancient human of the Novosvobodnaya archaeological culture dated to about 3,500 years before our era. The SNPs revealed during the analysis indicate that the mtDNA belongs to haplogroup V7, which is widely spread in modern Europeans and occurrs in cultures that used to exist in Central Europe. The current findings are consistent with the hypothesis that the Novosvobodnaya culture derived from early archaeological cultures of Northern and Central Europe and is now classified as an independent archaeological culture. However, this conclusion requires a thorough genetic analysis of samples from both the Novosvobodnaya and Maikop archaeological cultures. To date, there have been no available data on the status of the mtDNA haplogroup for the Maikop culture and, presumably, the ancestral Late Anatolian Eastern Chalcolithic period of phases III–IV (Amuk F).

## Abbreviations

mtDNA: mitochondrial DNA
SNP: single-nucleotide polymorphism
